# Breaking the Silence Associated With Death and Dying: New Directions in End-of-Life Research

**DOI:** 10.1093/geroni/igaa057.2227

**Published:** 2020-12-16

**Authors:** Sara Stemen, Peter Lichtenberg

## Abstract

Conversations surrounding end-of-life care and bereavement continue to remain relatively silenced within gerontology and the general population. The purpose of this symposium is to break the silence associated with death, dying, and bereavement by sharing emerging perspectives and interventions related to end-of-life experiences. This symposium features four presentations that examine bereavement and end-of-life care from the viewpoints of individuals, families, practitioners, and researchers. Carr provides a comprehensive overview of the current state of research regarding death, dying, and bereavement - mapping out how current technological and demographic shifts have changed the nature of end-of-life experiences. Stemen presents an illustrative case study that examines how cause of death (e.g., chronic illness, suicide) shapes grief and subsequent social relationships for surviving individuals. Utz explores conversations that occur between families and professionals embedded within the hospice system, showcasing reactions from families who experienced live discharge from hospice services. Last, Ogle sheds light on the roles taken on by state tested nursing assistants (STNAs) in end-of-life care as well as the training and education they receive and need on end-of-life issues. Lichtenberg, our discussant, will tie these emerging perspectives together in order to initiate an important dialogue with attendees regarding the actions needed to break the silence associated with death and dying so that we can better serve individuals, families, and professionals.

